# *Marsdenia tenacissima* Extract Induces Autophagy and Apoptosis of Hepatocellular Cells via MIF/mToR Signaling

**DOI:** 10.1155/2022/7354700

**Published:** 2022-03-04

**Authors:** Shuai Lin, Qianwen Sheng, Xiaobin Ma, Shanli Li, Peng Xu, Cong Dai, Meng Wang, Huafeng Kang, Zhijun Dai

**Affiliations:** ^1^Department of Oncology, The Second Affiliated Hospital of Xi'an Jiaotong University, Xi'an 710004, China; ^2^Department of Oncology, Xi'an International Medical Center Hospital, Xi'an, China; ^3^Department of Thyroid Breast Surgery, Xi'an International Medical Center Hospital, Xi'an, China; ^4^Department of Breast Surgery, The First Affiliated Hospital, College of Medicine, Zhejiang University, Hangzhou 310003, China

## Abstract

Hepatocellular carcinoma (HCC) seriously endangers humans. In traditional Chinese medicine, *Marsdenia tenacissima* (MTE) has anti-inflammatory, antiasthmatic, antihypertensive, and anticancer effects. This study reveals the antiproliferative effect of MTE on the HCC cells *in vitro* and provides a theoretical basis for the development and clinical application of anti-HCC agents. *Methods*. MHCC-97H and HepG2 cells were cultured *in vitro* and exposed to various concentrations and durations of MTE, and an MTT assay was used to detect the effects of MTE on cell proliferation. Transmission electron microscopy revealed the morphological changes in the two cell lines after MTE stimulation. The MTE effects on the apoptosis and cell cycle distribution of the cell lines were detected by flow cytometry. Western blotting and qRT-PCR were used to detect target gene expression at the protein and mRNA levels, respectively. *Results*. MTE reduced the viability of the MHCC-97H and HepG2 cells in a dose- and time-dependent manners (*P* < 0.05). Autophagic vesicles and apoptotic bodies were found in the MHCC-97H and HepG2 cells after MTE incubation, and the Annexin V-PI assay showed that the apoptotic rates of the cell lines increased with increasing MTE concentration (*P* < 0.05). Autophagy inducer rapamycin promoted the MTE-induced apoptotic rates of the cell lines, whereas autophagy inhibitor chloroquine inhibited the apoptotic rates. More cells in the S phase were found in the two cell lines after MTE treatment (*P* < 0.05). After MTE incubation, MIF, CD47, and beclin-1 protein levels significantly increased. Furthermore, in the MTE group, Akt, mTOR, and caspase3 expressions decreased; however, LC 3 expression increased, which was significantly different from the control group (*P* < 0.05). *Conclusions*. MTE inhibited proliferation and induced autophagy, apoptosis, and S phase cell cycle arrest in the MHCC-97H and HepG2 cells. These effects might be related to the activation of MIF and mTOR signaling inhibition.

## 1. Introduction

According to the GLOBOCAN statistics, in 2018, there were 841,000 new cases of hepatocellular carcinoma (HCC) and 782,000 HCC-related deaths worldwide, 48% of which were in China [1]. In recent decades, the incidence and mortality associated with HCC have been far ahead of those of many other cancers in China. It is the third most common cancer in China after lung and gastric cancer and ranks second in terms of mortality. Without timely intervention, people carrying hepatitis, especially hepatitis B, will develop cirrhosis, and eventually, HCC [2]. Additionally, aflatoxin, long-term heavy drinking, drinking polluted water, and genetic factors are also the risk factors for HCC [3]. Currently, HCC treatments are mainly based on surgical interventions and comprehensive therapies. However, because of the rapid progression of HCC, most patients are diagnosed in the middle and late stages of cancer when they seek medical attention, and only 20% of cases meet the surgical requirements [4]. Furthermore, low sensitivity to radiotherapy and chemotherapy, interventional therapy limitations, and a high recurrence rate mean that the five-year survival rate for HCC is low [5]. Therefore, exploring novel targeted drugs has become a top priority for medical practitioners.

Traditional Chinese medicine is one of the main methods used in many cancer therapies, for example, HCC therapy. Combined with surgery, radiotherapy, chemotherapy, interventional therapy, and targeted therapy, Chinese medicine plays a vital role by focusing on strengthening the body and eliminating diseases. *Marsdenia tenacissima* (MTE) is a common Chinese herbal medicine produced in Yunnan and Guizhou, and it contains complex ingredients, such as alkaloids, steroidal ester glycosides, organic acids, resins, and polysaccharides. It is used to treat asthma, bronchitis, and other diseases [6]. The discovery of other positive effects means that it has been available in a tablet form and as an injection reagent called Xiaoaiping since 1984 [7]. Previous research found that butanol extracts of MTE enhanced patient immunity, while ethyl acetate and petroleum ether extracts were toxic to tumor cells, thus exerting anticancer effects. Pharmacological studies have shown that new C21 steroid glycosides isolated from MTE induce the aggregation, assembly, and stabilization of cell microtubules and inhibit the mitosis of cancer cells, thereby inhibiting cancer growth [8]. In addition, compared to advanced rectal cancer patients receiving chemotherapy alone, patients treated with chemotherapy and MTE had a higher response rate (34.5% vs. 54.5%) and longer progression-free survival (6.43 vs. 7.97 months) [9]. Thus, MTE and its extractions are widely used as a monotherapy or as adjunctive therapy for gastric cancer, nonsmall cell lung cancer, esophageal cancer, and breast cancer [10–12]. However, there has been little basic or clinical research on MTE.

Apoptosis and autophagy are vital processes for cell death. Therefore, this study evaluated the effect of MTE on the proliferation, autophagy, apoptosis, and cell cycle distribution of two HCC cell lines (MHCC-97H and HepG2). The molecular mechanisms underlying MTE were also explored and elucidated.

## 2. Methods

### 2.1. Cells and Chemicals

The human HCC MHCC-97H and HepG2 cell lines were donated by other laboratories and cultured in Dulbecco's modified Eagle's medium (Gibco, Carlsbad, CA, USA), containing 10% fetal bovine serum (FBS; Gibco) and 0.1% penicillin-streptomycin solution (Gibco) in a humidified 5% CO_2_ environment at 37°C. MTE was purchased from Sanhome Pharmaceutical Limited Company.

### 2.2. Cell Proliferation Assay

A 3-(4,5)-dimethylthiahiazo (-z-y1)-3,5-di-phenytetrazoliumromide (MTT) assay was performed to explore the effect of MTE on HCC cell proliferation. In brief, the MHCC-97H and HepG2 cells were inoculated at a density of 8 × 10^3^ cells/100 *μ*L with five replicate wells in a 96-well plate and stimulated with various concentrations of MTE (0, 12.5, 25, 50, 100, 200, and 400 mg/mL) for 24 h, 48 h, and 72 h. After washing with phosphate-buffered saline (PBS), 20 *μ*L of MTT solution (5 mg/mL) was added to each well, and the plates were incubated for 4 h at 37°C in a humidified 5% CO_2_ incubator (Thermo Fisher Scientific, Waltham, MA, USA). Finally, after discarding the supernatant, 160 *μ*L of dimethyl sulfoxide (DMSO) was added to each well and the plates were shaken at low speed for 10 min. Optical density (OD) values were measured using a microplate reader at 490 nm.

### 2.3. Electron Microscopy

MTE-treated MHCC-97H and HepG2 cells were observed by electron microscopy to determine whether autophagy and apoptosis occurred. Firstly, MHCC-97H and HepG2 cells were incubated with 0, 35, and 50 mg/mL MTE for 48 h. After trypsinization and centrifugation, the cells were collected and fixed in 0.05 M cacodylate buffer (pH = 7.0) supplemented with 2.5% glutaraldehyde and 2.5% formaldehyde. Then, the cells were kept in 1% OsO4 cacodylate buffer for 24 h at 4°C, rinsed once with the same buffer, dehydrated with acetone, and embedded in epoxy resin. Ultrathin sections (70 nm) were obtained using a Leica Ultracut UCT ultramicrotome, stained with lead citrate and uranyl acetate, and observed using electron microscopy.

### 2.4. Cell Apoptosis Analysis

The MHCC97-H and HepG2 cells were treated with different concentrations of MTE, MTE plus the autophagy activator rapamycin (Rapa) (MCE, Monmouth Junction, NJ, USA), or an autophagy inhibitor (chloroquine [CQ]; Sigma-Aldrich, Saint Louis, MO, USA) for 48 h. Then, the cells in the supernatant and adherent cells were harvested and washed twice with PBS. The cells (1 × 10^6^) were suspended in 100 *μ*L binding buffer and stained with FITC-labeled Annexin-V (5 *μ*L) and propidium iodide (5 *μ*L) without light for 10 min. The apoptotic rate of the cells was examined by flow cytometry (FCM; Becton Dickinson and Company, East Rutherford, NJ, USA).

### 2.5. Cell Cycle Analysis

MTE-treated MHCC-97H and HepG2 cells were collected and fixed in cold 70% ethanol overnight at 4°C. Then, the cells were washed once with PBS and incubated with a staining working solution (RNase A: propidium iodide = 1 : 9) for 30 min in the dark at room temperature. Finally, the cell cycle distribution of the HCC cells was assessed using FCM.

### 2.6. Western Blotting (WB) Assay

The MHCC-97H cells were exposed to 0, 17.5, 35, and 70 mg/mL MTE for 48 h, and protein lysates were collected as described in our previous study [13]. The proteins were separated by 10% sodium dodecyl sulfate-polyacrylamide gel electrophoresis, transferred to polyvinylidene fluoride membranes, and then they blocked with Tris-buffered saline with 0.1% Tween 20 containing 5% bovine serum albumin for 2 h at room temperature. After treatment with primary antibodies at 4°C overnight, the membranes were incubated with secondary antibodies for 1 h at room temperature. The primary antibodies used in the WB assay were as follows: rabbit antimicrotubule-associated protein 1 light chain 3 I/II (LC3 I/II; 4108, Cell Signaling Technology, Danvers, MA, USA [CST]), rabbit anticaspase 3 (9662, CST), rabbit antimacrophage migration inhibitory factor (MIF) (87501, CST), rabbit anti-CD74 (63000, CST), rabbit antibeclin-1 (3495, CST), mouse anti-*β*-actin (3700, CST), rabbit antiserine/threonine kinase (Akt; 4691, CST), and rabbit antimammalian target of rapamycin (mTOR; 2983, CST).

### 2.7. Quantitative Real-Time Polymerase Chain Reaction (qRT-PCR)

The MHCC97-H and HepG2 cells were stimulated with various concentrations of MTE for 48 h. The total RNA content from the treated cells was extracted using TRIzol (Sigma, USA) and used to synthesize cDNA. The cDNA was subsequently used for PCR amplification to detect gene expression at the mRNA level. The 2^−ΔΔCt^ method was used to analyze the mRNA expressions of Akt, mTOR, LC 3, caspase 3, and *β*-actin. The sequences of the primers used are listed in Table 1.

### 2.8. Statistics

All experiments were repeated at least three times. The results are displayed as mean standard ± deviation ($\bar{x}$ ± SD) and were analyzed using SPSS software (version 21.0, IBM Corp., Armonk, NY, USA). Differences between multiple groups were compared by one-way analysis of variance (ANOVA), and the significant difference levels were ^*∗*^*P* < 0.05, and ^*∗∗*^*P* < 0.01.

## 3. Results

### 3.1. MTE Inhibits the Proliferation of MHCC-97H and HepG2 Cells

**Figure 1:**
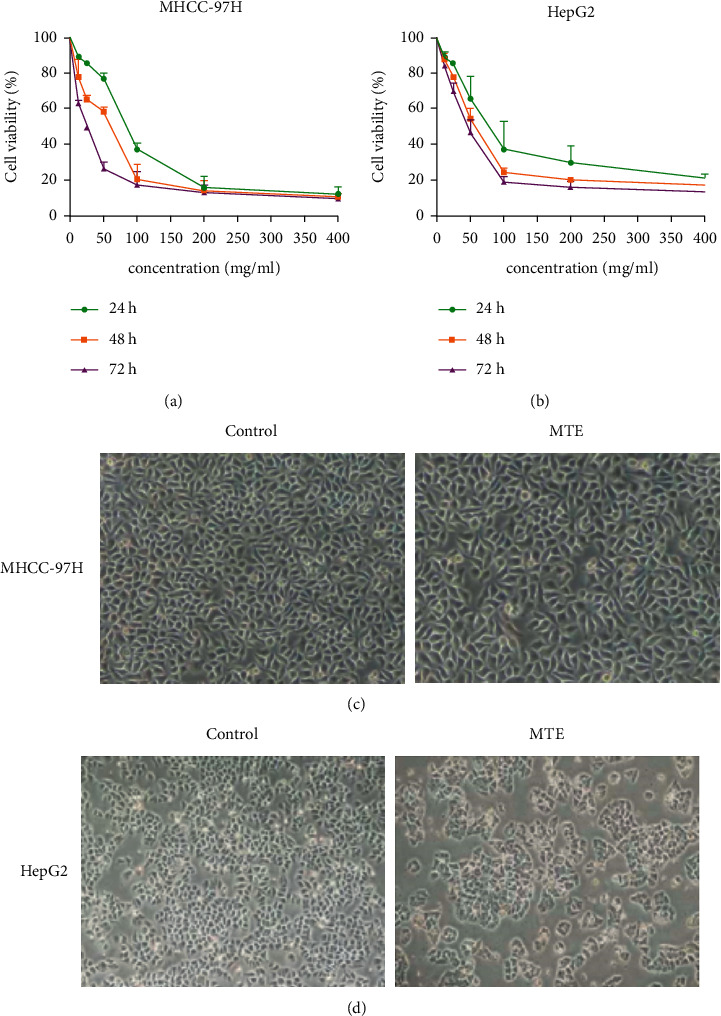
MTE suppressed the proliferation of the MHCC-97H and HepG2 cells in a dose- and time-dependent manner. (a, b) The MHCC-97H and HepG2 cells were stimulated with various concentrations of MTE (0, 12.5, 25, 50, 100, 200, and 400 mg/mL) for 24 h, 48 h, and 72 h. The cell viabilities of (a) the MHCC-97H and (b) HepG2 were assessed by MTT assay. (c) The cellular morphologies of the MHCC-97 cells in the control and MTE treatment group (35 mg/mL) were observed using electron microscopy (×40). (d) The cellular morphologies of the HepG2 cells in the control and MTE treatment group (50 mg/mL) were observed using electron microscopy (×40). *n* = 5.

The human HCC MHCC-97H cells with high invasive potential and the HepG2 cells with a low invasive potential were treated with various concentrations of MTE for different durations (24, 48, and 72 h). As shown in Tables 2 and 3, even at a low concentration (12.5 mg/mL), MTE treatment significantly decreased the viability of the MHCC-97H and HepG2 cells. In addition, the cell viability rates for the MHCC97-H and HepG2 cells decreased sharply as the MTE concentration and treatment time increased (Figures 1(a) and 1(b)). Furthermore, the MHCC-97H cells seemed more sensitive to MTE administration than HepG2 cells, as evidenced by the lower survival rate of the MHCC-97H cells under the same conditions.

The cellular morphology of the MHCC-97H and HepG2 cells was observed under a microscope after they had been treated with 35 mg/mL and 50 mg/mL MTE, respectively, for 48 h. The number of live cells declined in the treatment group compared to that of the control group, their cell volumes decreased, and the cells shrank and became rounded (Figures 1(c) and 1(d)). In addition, we observed cell debris and increased particles in the cytoplasm, and cell chromatin had gathered close to the nuclear membrane, which suggested pyknotic cell death in the MTE treatment group.

### 3.2. MTE Induces Autophagy and Apoptosis of MHCC-97H and HepG2 Cells

**Figure 2:**
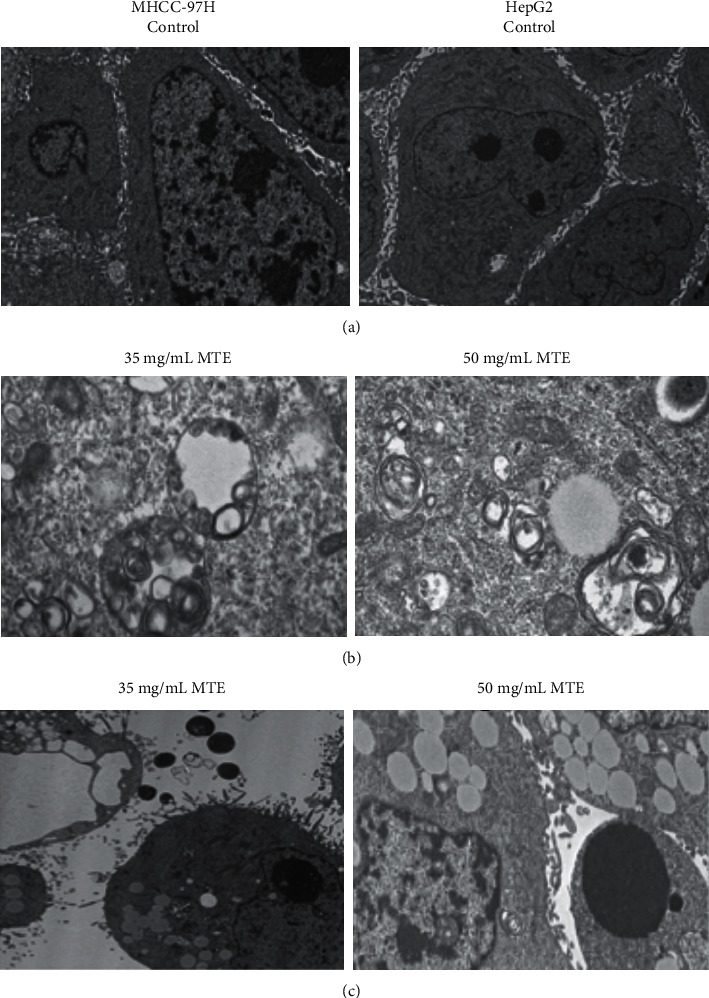
MTE induced autophagy and apoptosis of the MHCC-97H and HepG2 cells. (a) Normal morphologies of the MHCC-97H and HepG2 cells were observed using transmission electron microscopy. (b) 35 mg/mL and 50 mg/mL MTE treatment induced the formation of autophagosomes and autophagosome-lysosome structures in the MHCC-97H and HepG2 cells, respectively. (c) 35 mg/mL and 50 mg/mL MTE treatment induced the formation of apoptotic bodies in the MHCC-97H and HepG2 cells, respectively.

**Figure 3:**
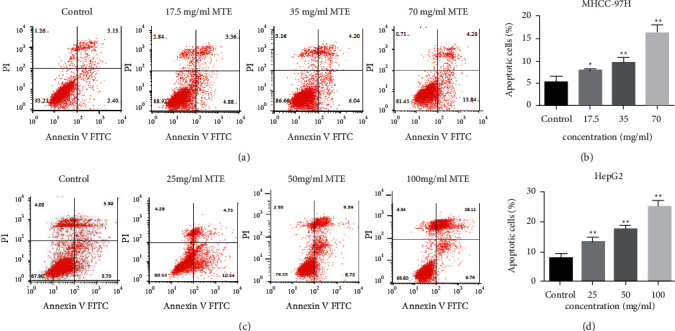
MTE induced apoptosis of the MHCC-97H and HepG2 cells in a dose-dependent manner. (a) Apoptotic rates for the MHCC-97H cells after MTE treatment (17.5 mg/mL, 35 mg/mL, and 70 mg/mL) were detected by FCM. (b) Quantitative data for (a). (c) Apoptotic rates for the HepG2 cells after MTE treatment (25 mg/mL, 50 mg/mL, and 100 mg/mL) were detected by FCM. (d) Quantitative data for (c). *n* = 3.

The MHCC-97H and HepG2 cells were observed using electron microscopy observations after they had been treated with 35 and 50 mg/mL MTE for 48 h, respectively. The results revealed that the chromatin in the MHCC97-H and HepG2 cells was evenly distributed in the control group (Figure 2(a)). MTE treatment induced the formation of autophagosomes and autophagosome-lysosome structures in the MHCC97-H and HepG2 cells (Figure 2(b)). Furthermore, apoptotic bodies were also observed in the treated cells (Figure 2(c)). An Annexin-V/PI assay was used to evaluate the apoptotic rates of the MHCC-97H and HepG2 cells induced by MTE treatment.  Figure 3 shows that the percentage of apoptotic MHCC-97H and HepG2 cells increased as the MTE concentration rose. The 17.5, 35, and 70 mg/mL MTE concentrations resulted in the apoptotic rates of 7.98 ± 0.30%, 9.72 ± 1.05%, and 16.7 ± 1.37% in the MHCC97-H cells, respectively, while 25 mg/mL, 50 mg/mL, and 100 mg/mL MTE led to the apoptotic rates of 13.46 ± 1.19%, 17.63 ± 0.99%, and 25.77 ± 1.14% in the HepG2 cells, respectively. Statistical differences were found between the MHCC-97H and HepG2 treatment and control groups.

**Figure 4:**
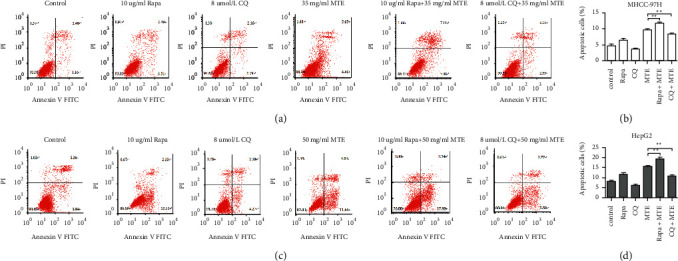
Relationship between autophagy and apoptosis induced by MTE treatment. (a) The MHCC-97H cells were treated with Rapa (10 *μ*g/mL), CQ (8 *μ*mol/L), MTE (35 mg/mL), Rapa (10 *μ*g/mL) plus MTE (35 mg/mL), or CQ (8 *μ*mol/L) plus MTE (35 mg/mL). The apoptotic cells were detected by FCM. (b) Quantitative data for (a). (c) The HepG2 cells were treated with Rapa (10 *μ*g/mL), CQ (8 *μ*mol/L), MTE (50 mg/mL), Rapa (10 *μ*g/mL) plus MTE (50 mg/mL), or CQ (8 *μ*mol/L) plus MTE (50 mg/mL). The apoptotic cells were detected by FCM. (d) Quantitative data for (c). *n* = 3.

To explore the relationship between autophagy and apoptosis induced by MTE treatment, the MHCC-97H and HepG2 cells were incubated with MTE, MTE combined with an autophagy activator (Rapa), or an autophagy inhibitor (CQ) for 48 h, and the apoptotic rates were examined by FCM.  Figure 4 shows that in the presence of MTE, Rapa increased the apoptotic rate of the two cell lines. However, additional CQ reduced the apoptotic rate. The above results suggest that MTE not only induces autophagy and apoptosis but also promotes apoptosis.

### 3.3. MTE Induces S Phase Cell Cycle Arrest in MHCC-97H and HepG2 Cells

**Figure 5:**
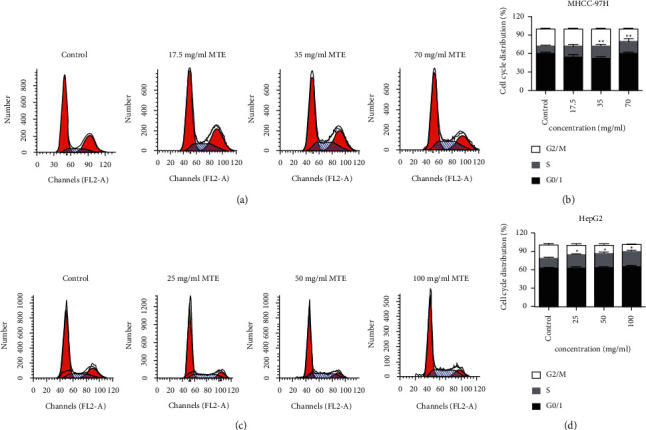
MTE induced the S cell cycle arrest in the MHCC-97H and HepG2 cells. (a) Cell cycle distributions of the MHCC-97H cells after MTE treatment (17.5 mg/mL, 35 mg/mL, and 70 mg/mL) were detected by FCM. (b) Quantitative data for (a). (c) The cell cycle distributions of the HepG2 cells after MTE treatment (25 mg/mL, 50 mg/mL, and 100 mg/mL) were detected by FCM. (d) Quantitative data for (c). *n* = 3.

The PI single staining assay was used to detect the cell cycle distribution of the two types of HCC cells. With MHCC-97H cells, the percentage of cells in the S phase increased remarkably from 12.55% to 17.78%, 20.74%, and 23.61% after 17.5, 35, and 70 mg/mL MTE stimulation, respectively (Figures 5(a) and 5(b)). The MTE treatment at doses of 25, 50, and 100 mg/mL caused 18.54%, 22.10%, and 24.62% of HepG2 cells to be arrested in the S phase, respectively (Figures 5(c) and 5(d)). Thus, the results showed that MTE might play a role by inducing the S phase cell cycle arrest.

### 3.4. MTE Induces the Autophagy of HCC Cells by Inhibiting the Akt/mTOR Pathway via MIF

**Figure 6:**
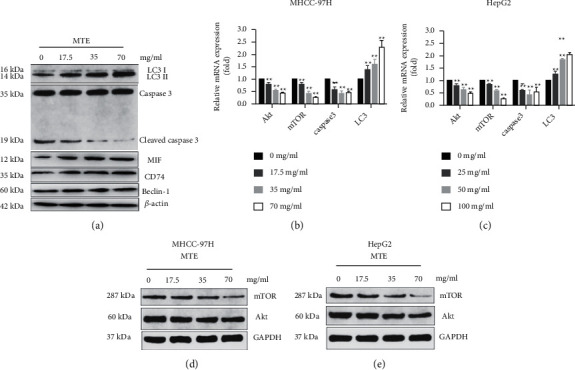
MTE induced the autophagy of HCC cells by inhibiting the Akt/mTOR pathway via MIF. (a) LC3 I, LC3 II, caspase3, cleaved caspase3, MIF, CD74, Beclin-1, and *β*-actin protein levels in MTE (17.5 mg/mL, 35 mg/mL, and 70 mg/mL)-treated MHCC-97H cells were examined by WB assay. (b) Akt, mTOR, caspase 3, and LC 3 mRNA levels in MTE (17.5 mg/mL, 35 mg/mL, and 70 mg/mL)-treated MHCC-97H cells were detected by qRT-PCR assay. (c) The mRNA levels for Akt, mTOR, caspase 3, and LC 3 genes in MTE (25 mg/mL, 50 mg/mL, and 100 mg/mL)-treated HepG2 cells were detected by qRT-PCR assay. (d) The mTOR, Akt, and GAPDH protein levels in MTE (17.5 mg/mL, 35 mg/mL, and 70 mg/mL)-treated MHCC-97H cells were detected by WB assay. (e) The mTOR, Akt, and GAPDH protein levels in MTE (17.5 mg/mL, 35 mg/mL, and 70 mg/mL)-treated HepG2 cells were detected by WB assay. *n* = 3.

Figure 6(a) shows that the increased expression of beclin-1 and the ratio of LC 3 II to LC3 I reflect the occurrence of autophagy, whereas the decreased ratio of cleaved caspase 3 to caspase 3 in MTE-stimulated MHCC-97H cells reflects the occurrence of apoptosis. Additionally, the protein expressions of MIF and its molecular receptor CD74 in the MHCC-97H cells significantly increased after MTE treatment (Figure 6(a)). MIF is reported to be an upstream regulator of the mTOR signaling pathway, and the mTOR pathway is now considered a target for cancer therapy. Therefore, the protein and mRNA levels of Akt and mTOR in the MHCC-97H and HepG2 cells were quantified by WB and qRT-PCR. The results revealed that an increase in the MTE concentration led to sharp decreases in the expressions of Akt and mTOR (Figures 6(b)–6(d)). Therefore, MTE-induced autophagy might be related to the inhibition of Akt/mTOR signaling via MIF.

## 4. Discussion

Autophagy is a normal cellular process in the body that provides a material basis for the development and maintenance of cellular homeostasis. Damaged or aged organelles and misfolded or mutated proteins are wrapped in the double-layer membrane of the rough endoplasmic reticulum, and the formed autophagosomes are fused with lysosomes to form a monolayer of autophagolysosomes to degrade these components [14]. Autophagy is closely related to cancer, and its effects on cancer are controversial. Autophagy promotes cell survival under stress conditions, such as hypoxia and pathogen infection [15]. Inhibiting autophagy suppresses the growth of pancreatic tumors *in vivo* and *in vitro* [16]. Hydroxychloroquine, an autophagy inhibitor, has been shown to increase tumor cell death alone or exert stronger tumor-killing potential in combination with other agents in preclinical studies [17]. Many drugs with the potential to induce autophagy have been widely used as therapeutic intervention agents during cancer treatment [18]. Yan et al. found that baicalein has an antibreast cancer effect by inducing autophagy in the breast cancer cells [19]. Nano-C60- and cysteamine-activated autophagy reverses the resistance of the MCF-7 cells to doxorubicin and enhances the efficacy of chemotherapy [20]. In this study, we found that MTE treatment induced the formation of autophagosomes and increased the mRNA level of the LC 3 gene in the MHCC-97H and HepG2 cells, indicating that MTE might suppress the HCC cell growth by inducing autophagy.

Apoptosis is the autonomous programmed death of cells. It is controlled by various genes and plays an important role in the growth and development of humans [21]. Drugs, such as chemotherapeutics, can induce the apoptosis of tumor cells to suppress tumor growth [22]. When apoptosis occurs, it is usually accompanied by changes in cell morphology, such as nuclear condensation, high chromatin condensation, marginalization, and chromosome breakage, and apoptotic bodies are usually found during the late stage of cancer progression. In our study, we found that MTE treatment induced the above-mentioned changes in cell morphology and apoptotic bodies after observing cells using an inverted microscope and a transmission electron microscope. The FCM assay further confirmed the occurrence of apoptosis in the MHCC-97H and HepG2 cells after MTE incubation. Previous studies have found that autophagy has a dual effect because it inhibits and promotes tumor growth under different conditions. Thus, we investigated the association between autophagy and apoptosis induced by MTE incubation. Combined with autophagy inducer (Rapa), MTE treatment resulted in more apoptotic cells than MTE alone, however, in the presence of an autophagy inhibitor (CQ), MTE treatment reduced the number of apoptotic cells compared to MTE alone, suggesting that MTE-induced autophagy promoted MTE-induced apoptosis, thereby exerting a strong inhibitory effect on the growth of HCC cells.

MIF was first discovered in the culture medium of active T lymphocytes and macrophages, and it mediates the innate and adaptive immune system to exert immune-regulatory functions [23]. Although recent studies have indicated that MIF is involved in the regulation of autophagy in tumor cells, its role is unclear [24, 25]. Silencing MIF reversed the conversion of autophagy marker LC 3-I to LC 3-II and induced mitochondrial autophagy [26]. However, recombinant human MIF increased autophagy in glioblastoma cells, and the knockout of endogenous MIF inhibited autophagy [27]. In breast cancer cells, MIF expression promotes autophagy, thereby inhibiting tumorigenesis and enhancing sensitivity to chemotherapeutic drugs [28]. In this study, we found that MTE treatment induced autophagy and elevated MIF and CD47 expressions in the HCC cells, indicating that MIF might participate in the MTE-stimulated autophagy process.

The phosphatidylinositol-4,5-bisphosphate 3-kinase (PI3K)-Akt-mTOR pathway not only regulates the growth, proliferation, differentiation, and apoptosis of normal cells but also regulates the occurrence and development of cancer and its sensitivity to different therapies [29, 30]. Akt and mTOR were found to be overexpressed in HCC tissues, and their expression was positively associated with the degree of malignancy [31]. In addition, normal cells can transform into HCC cells by activating mTOR signaling [32]. Drugs, such as buparlisib, everolimus, and perifosine, which target the PI3K/Akt/mTOR pathway, have the potential to inhibit survival pathways and induce apoptosis and autophagy in cancer cells, and they have been found to have favorable antitumor efficacies in clinical trials [33]. Similar to these results, we found that treatment of the MHCC-97H and HepG2 cells with MTE decreased the expressions of Akt and mTOR in a dose-dependent manner. These results suggested that MTE induced autophagy in the HCC cells by suppressing the PI3K/Akt/mTOR signaling pathway.

Endogenous MIF has been reported to be an upstream regulator of the mTOR pathway, and Zhang et al. found that homocysteine induced autophagy-activated cell death by promoting MIF secretion and reducing mTOR expression [34]. Consistent with this, we found that treatment of the MHCC-97H and HepG2 cells with MTE promoted the expression of MIF and reduced the expression of Akt and mTOR, indicating that MTE might inhibit mTOR signaling via MIF to induce autophagy in the HCC cells. However, follow-up studies are required to verify the association between MIF and the mTOR pathway.

## 5. Conclusion

In summary, MTE inhibited the proliferation of the MHCC-97H and HepG2 cells by inducing autophagy, apoptosis, and S phase cell cycle arrest, and these effects might be related to MIF/mTOR signaling. Thus, MTE might be a potential therapeutic agent for HCC.

## Figures and Tables

**Table 1:** Primers used in the experiment.

Gene		Primer sequence (5′-3′)
Akt	Forward	5′- CAGGATGTGGACCAACGTGA -3′
	Reverse	5′- AAGGTGCGTTCGATGACAGT -3′

mTOR	Forward	5′-CCACTGGTCTATGCCATCCC -3′
	Reverse	5′-AATGTAGGAGAACGTGGGGC -3′

LC3	Forward	5′- TTCCGAGTTGCTGACTGACC -3′
	Reverse	5′-CCCTTGTAGCGCTCGATGAT -3′

caspase3	Forward	5′-TGCTATTGTGAGGCGGTTGT -3′
	Reverse	5′- TCCAGAGTCCATTGATTCGCT -3′

*β*-Actin	Forward	5′- GACAGTCAGCCGCATCTTCT -3′
	Reverse	5′- GCGCCCAATACGACCAAATC -3′

**Table 2:** The effect of MTE on MHCC-77H cell proliferation ($\bar{x}\pm \text{SD}$).

Concentration (mg/mL)	24 h		48 h		72 h	
	OD	SR (%)	OD	SR (%)	OD	SR (%)
0	0.346 ± 0.157	100	0.337 ± 0.122	100	0.448 ± 0.272	100
12.5	0.300 ± 0.142	86.5^∗^	0.261 ± 0.129	73.3^∗∗^	0.294 ± 0.169	67.4^∗∗^
25.0	0.286 ± 0.138	82.3^∗^	0.207 ± 0.099	58.6^∗∗^	0.214 ± 0.137	46.1^∗∗^
50.0	0.255 ± 0.116	73.9^∗∗^	0.169 ± 0.107	44.5^∗∗^	0.117 ± 0.052	29.9^∗∗^
100.0	0.142 ± 0.066	40.4^∗∗^	0.057 ± 0.009	18.4^∗∗^	0.066 ± 0.019	19.1^∗∗^
200.0	0.060 ± 0.034	17.6^∗∗^	0.040 ± 0.006	13.1^∗∗^	0.055 ± 0.016	16.0^∗∗^
400.0	0.041 ± 0.019	11.9^∗∗^	0.033 ± 0.011	9.8^∗∗^	0.044 ± 0.020	11.2^∗∗^

OD: optical density, SR: survival rate, ^∗^*P* < 0.05, and ^∗∗^*P* < 0.01.

**Table 3:** The effect of MTE on HepG2 cell proliferation ($\bar{x}\pm \text{SD}$).

Concentration (mg/mL)	24 h		48 h		72 h	
	OD	SR (%)	OD	SR (%)	OD	SR (%)
0	0.348 ± 0.052	100	0.344 ± 0.068	100	0.344 ± 0.024	100
12.5	0.310 ± 0.041	89.3^∗^	0.303 ± 0.053	88.5^∗^	0.292 ± 0.021	84.7^∗^
25.0	0.299 ± 0.045	86.1^∗^	0.270 ± 0.051	78.5^∗^	0.241 ± 0.007	70.3^∗∗^
50.0	0.230 ± 0.023	66.9^∗^	0.185 ± 0.022	54.6^∗∗^	0.162 ± 0.006	47.3^∗∗^
100.0	0.147 ± 0.042	42.9^∗∗^	0.083 ± 0.012	24.4^∗∗^	0.065 ± 0.0003	19.0^∗∗^
200.0	0.105 ± 0.014	30.9^∗^	0.070 ± 0.011	20.4^∗^	0.055 ± 0.004	16.1^∗^
400.0	0.080 ± 0.017	23.1^∗^	0.059 ± 0.010	17.4^∗^	0.046 ± 0.000	13.5^∗^

OD: optical density, SR: survival rate, ^∗^*P* < 0.05, and ^∗∗^*P* < 0.01.

## Data Availability

The data analyzed are available from the corresponding authors on reasonable request. Because of some confidential reasons, the participants of this study did not agree for their data to be shared publicly, and all authors do not wish to share the data with the database online before publication. Hence, supporting data is not available. However, the data used to support the findings of this study are available from the corresponding author upon reasonable request.
